# Direct comparison of activation maps during galvanic vestibular stimulation: A hybrid H_2_[^15^ O] PET—BOLD MRI activation study

**DOI:** 10.1371/journal.pone.0233262

**Published:** 2020-05-15

**Authors:** Sandra Becker-Bense, Frode Willoch, Thomas Stephan, Matthias Brendel, Igor Yakushev, Maximilian Habs, Sibylle Ziegler, Michael Herz, Markus Schwaiger, Marianne Dieterich, Peter Bartenstein

**Affiliations:** 1 Department of Neurology, University Hospital, Ludwig-Maximilians-Universität München, Munich, Germany; 2 German Center for Vertigo and Balance Disorders (DSGZ), University Hospital, Ludwig-Maximilians-Universität München, Munich, Germany; 3 Department of Nuclear Medicine, University Hospital, Ludwig-Maximilians-Universität München, Munich, Germany; 4 Institute of Basic Medical Sciences, University of Oslo, Oslo, Norway; 5 Department of Nuclear Medicine, Technical University, Munich, Germany; 6 Munich Cluster of Systems Neurology – SyNergy, Munich, Germany; McLean Hospital, UNITED STATES

## Abstract

Previous unimodal PET and fMRI studies in humans revealed a reproducible vestibular brain activation pattern, but with variations in its weighting and expansiveness. Hybrid studies minimizing methodological variations at baseline conditions are rare and still lacking for task-based designs. Thus, we applied for the first time hybrid 3T PET-MRI scanning (Siemens mMR) in healthy volunteers using galvanic vestibular stimulation (GVS) in healthy volunteers in order to directly compare H_2_^15^O-PET and BOLD MRI responses. List mode PET acquisition started with the injection of 750 MBq H_2_^15^O simultaneously to MRI EPI sequences. Group-level statistical parametric maps were generated for GVS vs. rest contrasts of PET, MR-onset (event-related), and MR-block. All contrasts showed a similar bilateral vestibular activation pattern with remarkable proximity of activation foci. Both BOLD contrasts gave more bilateral wide-spread activation clusters than PET; no area showed contradictory signal responses. PET still confirmed the right-hemispheric lateralization of the vestibular system, whereas BOLD-onset revealed only a tendency. The reciprocal inhibitory visual-vestibular interaction concept was confirmed by PET signal decreases in primary and secondary visual cortices, and BOLD-block decreases in secondary visual areas. In conclusion, MRI activation maps contained a mixture of CBF measured using H_2_^15^O-PET and additional non-CBF effects, and the activation-deactivation pattern of the BOLD-block appears to be more similar to the H_2_^15^O-PET than the BOLD-onset.

## Introduction

Brain activation techniques using positron emission tomography (PET) with ^15^O-labeled water were established in the early 1980s, introducing the era of functional imaging. H_2_^15^O-PET has been validated as a measure of regional cerebral blood flow (rCBF) [1], and is still considered to be the ‘‘gold standard” for rCBF measurements in humans, either as a quantitative (requiring arterial blood sampling) or as a non-quantitative technique delivering measure of rCBF as the surrogate marker of brain activity [2,3]. RCBF is defined as the flow of blood delivered per minute per unit volume of tissue, and relates to both brain metabolism and function.

Since magnetic resonance imaging (MRI) using blood-oxygenation-level-dependent (BOLD) was introduced in the 1990s, it has developed rapidly and has more or less taken over the domain of functional imaging, delivering high-sensitivity and relatively high spatial and temporal resolution without radioactive burden [4–6]. BOLD is a non-quantitative technique measuring a composition of not only rCBF, but also blood oxygenation and blood volume [7]. Rare comparative studies of H_2_^15^O-PET and BOLD MRI in humans reported that BOLD is positively correlated with rCBF (visual checkerboard stimulation) [8] and negatively with oxygen extraction fraction in PET (hand motor task) [9]. An animal study applying whisker stimulation in rodents reported a lowered number of activated voxels in H_2_^15^O PET compared to BOLD-MRI, and a difference in the activation centers in both the shape and location between PET and MRI [10]. However, these task-based H_2_^15^O-PET–MRI comparisons were so far acquired in separated successive scanning sessions.

A direct comparison minimizing temporal, physiologic, and functional variations by simultaneous PET and MRI measurements became feasible with the first generation of integrated hybrid scanners in 2010 [review 2]. Up to now, there are only a few publications available directly comparing CBF measurements with H_2_^15^O PET and MR with arterial spin label measures (ASL), but at baseline conditions without stimulation [2]. ASL is a non-invasive functional MRI technique that uses radiofrequency (RF)-labeled arterial blood as an intrinsic tracer and provides quantitative measurement of brain perfusion. Although ASL is assumed to be more similar to rCBF in H_2_^15^O-PET, the studies found significant rCBF differences between the two modalities despite an overall similarity [piglets 11; stroke patients 12; healthy humans 13]. Studies in humans directly comparing BOLD and H_2_^15^O-PET in a hybrid scanner under brain stimulation conditions are still lacking. This is most probably due to the difficulty of adequately adapting the technical and statistical prerequisites to a feasible and useful procedure. The aim of the current study was the simultaneous acquisition of BOLD and H_2_^15^O PET under a sensory stimulation condition (task-based design).

Since the 1990s, the vestibular system, which is involved in perception, ocular motor, postural, and vegetative control, has been explored by a number of unimodal PET and fMRI imaging studies applying different vestibular stimuli, e.g., caloric, galvanic (GVS), vestibular-evoked myogenic potentials (VEMP) or visual-motion stimulation, first in healthy subjects and later on in patients with distinct vestibular lesions [14–16,17]. Despite considerable variations in imaging modalities and stimulation techniques, a relatively consistent bilateral cortical and subcortical network of several distinct, mostly temporo-parietal areas could be identified, e.g., the posterior insula and retroinsular region, the superior temporal gyrus, the supramarginal gyrus, the inferior parietal lobule, the inferior frontal gyrus, the anterior cingulate gyrus, the anterior insula, and the hippocampus [17–21]. Its core region, corresponding to the parietoinsular vestibular cortex (PIVC) in primates, is located in the (retro)insular-opercular region [22–25]. The central vestibular system has been shown to be bilaterally organized [26,27], but characterized by a right-hemispheric predominance in right-handers and a left-hemispheric predominance in left-handers [23,28,29]. This lateralization has been repeatedly confirmed, especially by PET [30–35] and fNIRS [36] rather than fMRI studies mainly using caloric irrigation of the semicircular canals [37] or otolith stimulation by VEMPs [21,38].

H_2_^15^O-PET-studies were the first to describe simultaneous deactivations within the visual and somatosensory systems of both hemispheres during vestibular caloric irrigation of the semicircular canals in healthy volunteers showed [39,40]. This led to the concept of a reciprocal inhibitory cortical interaction between the two sensory systems, the visual and the vestibular [19,41,42]. However, the relatively constant vestibular activation-deactivation patterns varied in their weighting and expansiveness between the different imaging and stimulation modalities. Since vestibular stimulation paradigms are very well established in both PET and MRI, we consider vestibular stimulation an ideal paradigm for further investigations using hybrid PET-MRI. Because of its dual applicability in MRI and PET and its on-off character, we chose GVS for our hybrid H_2_^15^O-PET and BOLD MRI study on the vestibular system. GVS stimulates both vestibular semicircular canal and otolith afferents [43–48]. In humans it induces a sensation of being tilted or nudged most notably at stimulus onset [44–49]. GVS in healthy volunteers has been shown to consistently activate the human central vestibular network in both hemispheres [18,20,50–54].

The objectives of this study in healthy subjects were twofold:

1. To prove for the first time the feasibility of hybrid H_2_^15^O-PET and BOLD MRI scanning in a vestibular stimulation paradigm, e.g. using GVS.
2. To look for similarities and differences in BOLD signal increases and decreases (block as well as event-related responses) by direct comparison with the rCBF of H_2_^15^O-PET data in general (e.g., corresponding and/or contradicting signal changes), and with special regard to hemispheric laterality aspects and visual-vestibular interaction patterns.

## Material and methods

### Subjects

Twenty-one subjects (9 female) were recruited by word of mouth and newspaper advertisements between 2013 and 2017. The inclusion criteria required subjects to be older than 30 years and to be free of neurological, especially vestibular impairments, and medical treatment affecting the brain. Further exclusion criteria were the standard contradictions for MRI scanning, and pregnancy. All subjects underwent a detailed clinical neurological examination with special attention to functioning of the vestibular (e.g., Halmagyi head-impulse test, stance and gait), ocular motor (e.g., saccades, smooth pursuit etc.), somatosensory, and visual systems (clinical eyesight test) prior to inclusion. The data of two subjects had to be excluded from data analyses, one due to stimulator malfunction, and one due to an anatomical variation that precluded normalization to the MNI space. Therefore, the data from 19 subjects (mean age 47 +/- 10 years, 8 female) was included; 17 were right-handed (14 x 100%; 2 x 80%; 1 x 60%), and 2 were left-handed (-85.7%; -100%) [55,56]. The two left-handed volunteers were included in the analyses to maintain the statistical power. Since the acquisitions were simultaneous for PET and MRI, handedness should not lead to any systematic bias between the two methods.

The study was conducted according to regulations of the Helsinki Declaration and was approved by the local Ethics Committee of the Ludwig-Maximilians-Universität München, Germany (90–15), and the Radiation Protection Authorities (BfS Z5-22461/2-2015-002). All subjects gave their informed written consent.

### Data acquisition

**Fig 1:**
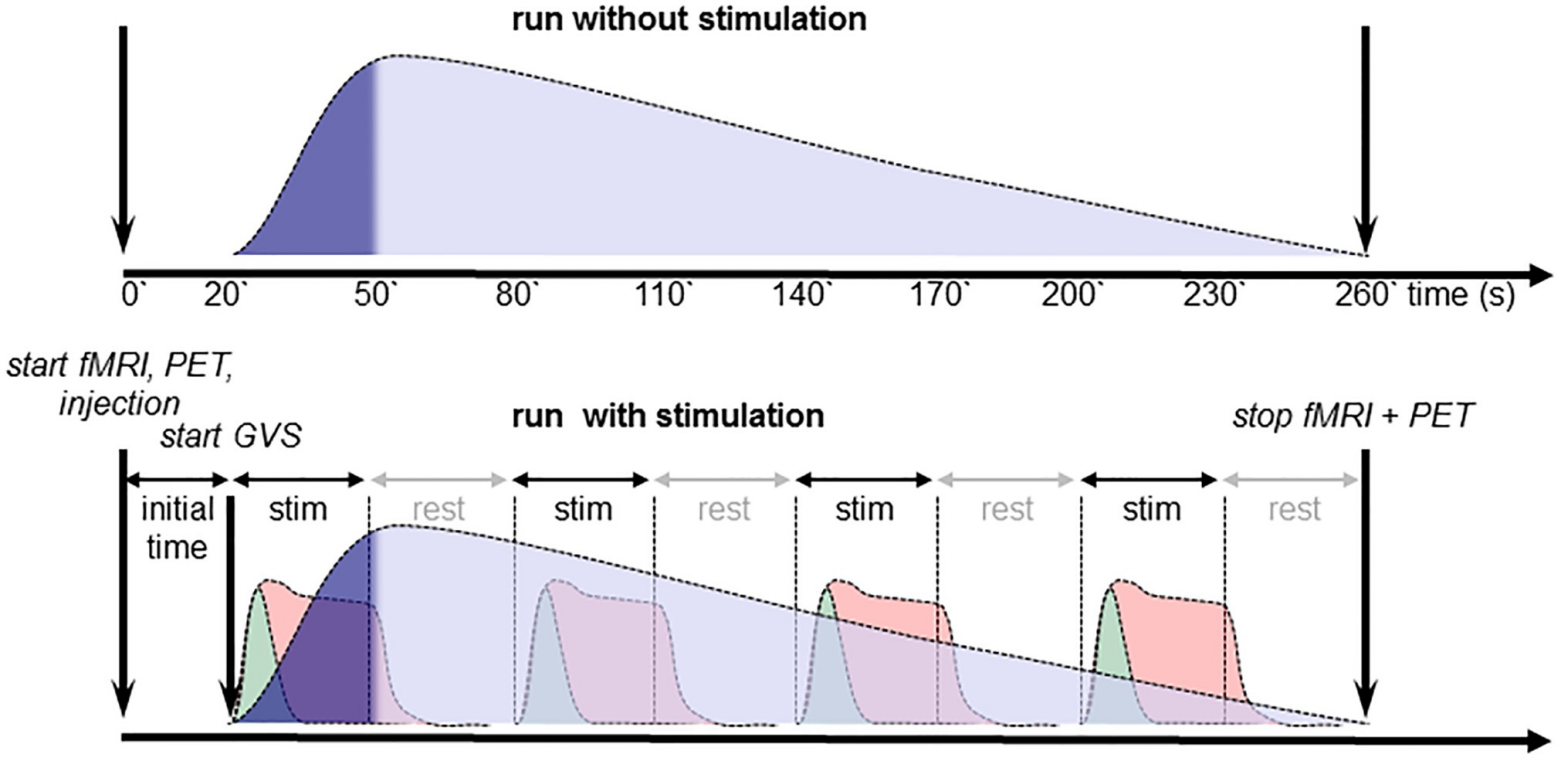
Experimental procedure. Simultaneous PET-MRI data acquisition protocol and depiction of the expected BOLD-responses, BOLD-onset (green), BOLD-block (red), and H_2_^15^O-PET activity curve (blue). PET data has been reconstructed and analyzed for the time periods depicted in dark blue. Imaging runs with (top) and without stimulation (bottom) were conducted in a randomized order.

Imaging data acquisition was performed on a hybrid PET-MR scanner (Siemens mMR) at a field strength of 3T [57]. Eight simultaneous PET-MRI runs were acquired from every subject in randomized order, four runs with stimulation and four runs at rest (Fig 1). In nine subjects, only six PET-MR runs (three rest and three stimulation runs) could be performed due to the long scanning procedure.

#### MRI

Functional MRI data were acquired using a combined 16-channel head-neck coil and an echo-planar imaging sequence (EPI, voxel-size 3.1x3.1x3.0 mm, 39 slices, interleaved acquisition, no slice gap, 64x64 voxels, TE 30 ms, TR 2500 ms, 101 consecutive image volumes per run). Initial dummy scans were discarded to account for T1 saturation effects of the MRI signal. An additional high-resolution T1 weighted structural scan (isotropic voxel size of 1 mm) was acquired from every subject using an MPRAGE sequence.

#### PET

750 MBq H_2_^15^O was administered intravenously slowly over 30 s using an infusion pump followed by immediate flushing with isotonic NaCl-solution. A 7-minute list-mode PET acquisition started at the same time as the injection. Data were reconstructed in a 2-step procedure on an offline workstation (e7 tool, Siemens). First, reconstruction was performed with short initial times frames (12 x 5 sec, 6 x 10 sec, 10 x 30 sec) to identify the slope of increase on the time activity curve in the brain (excluding slices from the neck). Across all individuals and scans there was a consistent slope of 30 seconds, and these 30 seconds were used in the second and final iterative reconstruction (OSEM algorithm with 3 iterations and 24 subsets) [58,59]. Attenuation maps were calculated using the T1 MPRAGE images in a CT and attenuation map synthesis algorithm based on a multi-atlas information propagation scheme [60]. The reconstruction resulted in a 256x256x127 matrix with voxel size of 1.0x1.0x2.0 mm.

### Experimental procedure

Subjects were placed supine inside the scanner wearing suitable hearing protection and had their eyes closed during data acquisition. We used an inflatable helmet-like pillow (crania ^™^, http://www.pearl-technology.ch/de/radiologie/crania) to minimize head motion. During the stimulation runs, galvanic vestibular stimulation with alternating current (AC-GVS) at a frequency of 1 Hz was applied in a block design [20]. Stimulation currents were generated using a stimulator built in-house that was controlled by a laptop computer running Matlab (R2009b, The Mathworks Inc.), and applied using rubber electrodes. The battery-driven stimulator was placed inside the Faraday cage of the scanner and controlled via fiber-optic data transmission. Electrodes were attached over both mastoid processes after skin preparation with Ten20 electrode gel, and secured with a head bandage. Prior to imaging, we applied a test stimulus to every subject to adjust the current strength to an individual minimal level that induced a clear vestibular sensation but no skin irritation. The applied current strengths were 2.5 mA (11 subjects), 2.25 mA (1 subject), 2 mA (4 subjects), and 1.75 mA (3 subjects). In order to minimize cutaneous sensations at the electrode site induced over time by the repetitive GVS [18,51], the skin was locally anesthetized with lidocaine gel. Lidocaine is known to modulate nociceptive tonic A-delta and C fiber discharges und thereby suppresses pain-induced brain activation, e.g. within the multisensory thalamus. However, we effects of lidocaine use on vestibular afferents are not expected. In this experimental procedure all volunteers consistently reported a feeling of being tilted or nudged every time the current was switched on, which was most intense at the beginning of each stimulation block, but continued mildly over the whole stimulation period. A metallic taste side effect was reported by 10 subjects in 27 out of 67 stimulation runs.

Half of the imaging runs were conducted as rest-runs without GVS; the order of rest- and stimulation-runs was randomized. However, the first data acquisition of every scanning session was a rest-run to measure the individual time interval between tracer injection and its arrival in the subject’s brain indicated by the increase of the count detection of the PET scanner. This time interval was used in the subsequent scans for optimal timing of the GVS start with the activity increase in PET.

During each stimulation run we applied 4 blocks of 1Hz AC-GVS. Stimulation lasted for 30 s per block and was followed by a rest block of 30 s. The onset time of the first stimulation block was adapted on the basis of the observed onset of tracer accumulation in the brain to ensure that stimulation did not start before PET data could be acquired. This shift of the time of the first stimulation onset shifted the whole block-design as well and therefore individually influenced the lengths of only the rest periods at the beginning and end of the imaging run. The inter-stimulus interval was not affected by this adaptation. PET data acquisition, MRI scanning, and the tracer injection pump were started simultaneously (Fig 1).

### MRI data analysis

We used SPM12 software and Linux workstations for imaging data processing (SPM v6906, http://www.fil.ion.ucl.ac.uk/spm/software/spm12/, Matlab R2018a). All MRI image volumes were corrected for head motion by realignment to the mean image of the respective run. Every subject’s structural scan was coregistered to the mean image of the realigned EPI images, followed by segmentation using the CAT12 toolbox in SPM (CAT12 version 1184, http://dbm.neuro.uni-jena.de/cat/). The parameters obtained by the segmentation process were then applied to warp all functional and structural images to the Montreal Neurological Institute (MNI) standard space. Functional data were smoothed using a Gaussian kernel of 12mm FWHM before statistical analysis.

Single-subject (first level) statistical analysis was performed using a general linear model (GLM) to assess the effects of GVS on the BOLD signal from the stimulation runs. For every subject, we constructed a first-level GLM that included a hemodynamic model of the stimulation blocks. Because we know from Stephan et al [52] that GVS-induced activations can be separated into onset-related and continuous components, we included an additional regressor that models hemodynamic responses to the onset events of the stimulation, i.e. single events without a temporal duration. To test for effects that decline or increase with each stimulation block, further regressors were included to model the linear increase of responses to onset and block with time. The first level model included a high pass filter with a cutoff at 120s; serial correlations were accounted for using an autoregressive AR(1) model during parameter estimation. Linear contrasts were defined to assess the effects related to the specific regressors. These contrasts were ‘BOLD-onset’, ‘BOLD-block, ‘BOLD-onset x time’, ‘BOLD-block x time’, each of which assessed the respective columns of the design matrix. First level contrast images were computed for all of these contrasts. To test for effects on the group level, the single-subject contrast images were entered into a 2nd-level model. Positive and negative effects for the different contrasts were estimated using one-sample t-tests. Additionally, we entered first level contrast images into paired t-tests to compare the effects of PET versus BOLD-onset, and PET versus BOLD-block on the group level. Results exceeding a threshold of p<0.05 corrected for multiple comparisons using the method of false discovery rate (FDR) [61] and a cluster size of more than 5 voxels are considered significant. The resulting regions were identified using the Juelich Histological and Harvard-Oxford Structural atlases [62,63] as included in the software FSLEyes released 2018 (https://git.fmrib.ox.ac.uk/fsl/fsleyes/fsleyes/), as well as the Hammers atlas that is included in the CAT12 toolbox. In large clusters comprising several brain areas, additional maxima closest to the maxima in the other modality were identified with SPM. They are indicated by * in the Tables. To compare peak results between the different imaging modalities, we extracted the contrast estimates as a correlate of the effect strength at the locations of selected cluster maxima.

### PET data analysis

To correct for head motion, every PET scan was coregistered to the mean-EPI of the MRI data from the respective subject. Following coregistration, the parameters obtained by the segmentation of the subject’s structural image were applied to warp the PET data to MNI space, followed by smoothing with a Gaussian kernel of 12 mm FWHM. Single-subject (first level) statistical analysis was performed using a general linear model to compare the PET images from the stimulation runs against the images from the rest runs. We applied global mean scaling by proportional scaling to account for differences in global signal between scans [3]. Global intracerebral signal calculation was based on a mask that excluded the area of the extra-cerebral arteries. We calculated the first level contrast GVS-rest using GLM analysis. To test for positive and negative effects on the group level, the single-subject contrast images were entered into a 2nd-level GLM that implemented a one-sample t-test. Results exceeding a threshold of p<0.05 corrected for multiple comparisons (FDR) [61] and a cluster size of more than 5 voxels are considered significant.

### Similarity analysis of activations and deactivations

To compare the extent and overlaps of activation maps between PET, BOLD-onset, and BOLD-block we calculated the Jaccard index between these maps. The Jaccard index for the two maps A and B has a range from zero to one, with an index of zero if A and B share no overlapping voxels, and an index of one if A and B cover identical voxels. Given the number of voxels in A and B as |A| and |B|, respectively, and the number of overlapping voxels as |A ⋂ B|, the Jaccard index J(A,B) is calculated by

$$J(A,B)=\frac{\left|A\cap B\right|}{\left|A\cup B\right|}=\frac{\left|A\cap B\right|}{\left|A|+|B|-|A\cap B\right|}$$

We calculated the Jaccard index for PET versus BOLD-onset, PET versus BOLD-block, and BOLD-onset versus BOLD-block, each of these maps was thresholded at p<0.05 (FDR).

### Lateralization analysis

We computed lateralization indices (LI) to describe the asymmetry of the reported activation maps. Computations were performed using the SPM-toolbox LI-tool [64] using total voxel values and no further thresholding by the toolbox. Mask images provided with the toolbox were used to exclude voxels located in the cerebellum and voxels within +/- 5 mm along the midsagittal plane. Thresholded activation and deactivation maps (p<0.05 FDR) were used as input images for LI computations. We computed whole-brain LI between the hemispheres, and LI for selected regions of interest (ROIs). The ROIs were entered into the toolbox as ROI-mask images. The ROI-mask images were defined as follows: insular-opercular ROI was extracted from the anatomy toolbox [62] by merging the areas OP1/OP2/OP3/OP4, cingulate sulcus visual (CSv) ROI was defined by spheres with a radius of 10mm around the MNI-coordinates x/y/z = -10/-26/41 and 9/-24/44, as given by Smith et al [65]. A ROI in frontal eye fields (FEF) was defined by spheres (radius 10mm) around the maximum coordinate obtained by the PET analysis in this region (44/-6/50 and -44/-6/50). Further, ROIs for the supplementary motor area (SMA) and the precuneus were defined by extracting these regions from the Harvard-Oxford Cortical Structural Atlas [63] at an atlas probability threshold of 20%. An ROI for the thalamus was extracted from the parcellation provided by Tzourio-Mazoyer [66]. As defined by the LI-tool, LI values are negative for lateralization towards the right hemisphere, positive for lateralization towards the left hemisphere.

## Results

### Main effects of vestibular stimulation

**Fig 2:**
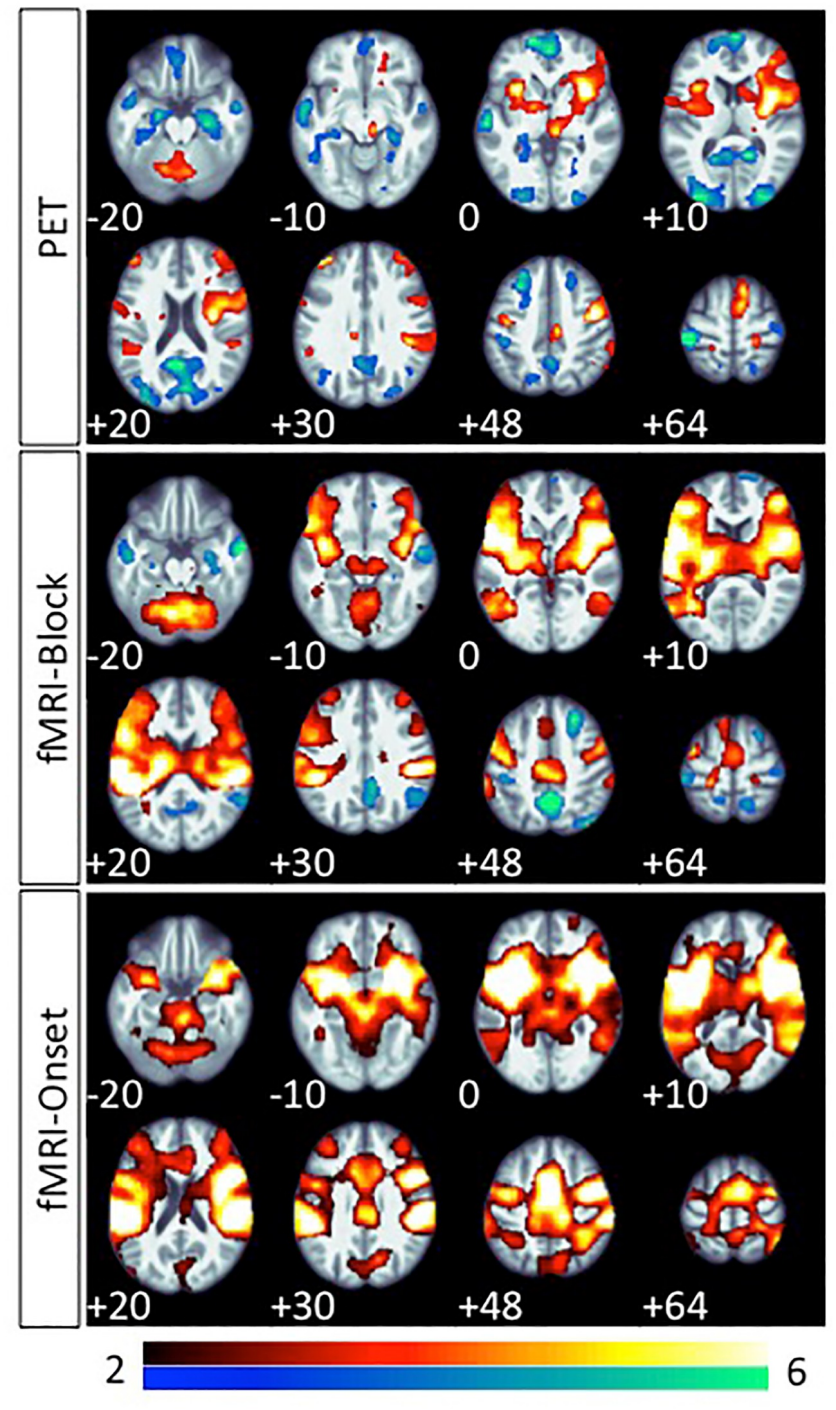
Group effects of vestibular galvanic stimulation. Main group effects of vestibular galvanic stimulation (GVS) compared to the rest condition in H_2_^15^O-PET, BOLD-block, and BOLD-onset (paired t-test; FDR < 0.05) superimposed onto a mean T1 image of the subject group. GVS activations (GVS vs. rest) are indicated in red, GVS deactivations (rest vs. GVS) in blue.

Overall, the group contrasts for rCBF PET, BOLD-block and BOLD-onset all exhibited significant bilateral activations in corresponding areas known to be involved in central vestibular processing and ocular motor function (Fig 2, Table 1). Thus, GVS seems to provide reliable activation patterns for use in further comparison of these contrasts.

**Table 1:** Group contrasts for rCBF PET, BOLD-block and BOLD-onset.

**Activations**	**PET**						**MRI-block**						**MRI-onset**					
	**x**	**y**	**z**	**T-value**	**Atlas**		**x**	**y**	**z**	**T-value**	**Atlas**		**x**	**y**	**z**	**T-value**	**Atlas**	
**Insula**	34	14	-2	9.21	R	anterior Insula	42	10	0	7.00	R	anterior insula	34	14	-2	9.73	R	anterior insula*
	32	-6	16	6.95	R	anterior insula	32	-6	16	4.72	R	anterior insula	38	-6	12	11.85	R	insula
							38	-10	-4	8.41	R	posterior insula	38	-10	-4	6.54	R	posterior insula*
	-32	14	2	6.83	L	anterior insula	-40	2	-4	8.15	L	anterior insula	-34	16	2	8.79	L	anterior insula
	-40	-4	12	4.52	L	insula	-36	-2	12	5.61	L	insula	-38	-4	12	9.10	L	insula
							-36	-16	-2	5.87	L	posterior insula	-38	-10	4	8.86	L	posterior insula
**Midline cingulate and frontal lobes**													4	-2	44	8.46	R	anterior cingulate g.
													0	34	14	3.56		anterior cingulate g.
													6	-28	28	5.31	R	posterior cingulate g.
	8	-22	50	5.37	R	precentral gyrus, medial part (CSv)	8	-28	52	6.06	R	precentral g., cingulate sulcus (CSv)	8	-22	50	5.22	R	precentral g., cingulate sulcus (CSv)*
							-6	-22	48	5.33	L	precentral g., cingulate sulcus (CSv)	-6	-22	48	4.87	L	precentral g., cingulate sulcus (CSv)*
							-10	-18	44	6.18	L	cingulate sulcus (CSv)	-10	-14	42	7.06	L	cingulate sulcus (CSv)
	8	0	76	5.33	R	sup. frontal g. (SMA)	4	-2	74	4.72	R	sup. frontal g. (SMA)	8	-2	58	6.64	R	sup. frontal g. (SMA)
	12	18	66	5.91	R	sup. frontal g. (SMA)												
	-6	0	72	3.65	L	sup. frontal g. (SMA)*												
							-2	-12	56	5.10	L	sup. frontal g. (SMA)	-2	-12	56	5.1	L	sup. frontal g. (SMA)
							-10	30	62	4.02	L	sup. frontal g. (SMA)						
							-6	16	48	3.17	L	sup. frontal g. (SMA)	-6	16	48	5.03	L	sup. frontal g. (SMA)
**Lat. frontal lobes**	40	52	26	4.98	R	middle frontal g.	40	44	24	3.91	R	middle frontal g.	38	40	26	4.09	R	middle frontal g.
	38	40	8	5.15	R	inferior frontal g.	40	34	12	5.11	R	inferior frontal g.	48	44	12	5.80	R	frontal pole
	52	12	4	3.57	R	inferior frontal g. opercular part*	52	12	4	7.74	R	inferior frontal g., opercular part	52	10	6	10.14	R	inferior frontal g., opercular part
	44	-6	50	7.10	R	precentral g.(FEF)	46	-4	52	5.48	R	precentral g. (FEF)	44	-2	52	9.53	R	precentral g. (FEF)
	-34	48	32	5.90	L	frontal pole	-38	36	14	6.07	L	Inf.r/middle frontal g.	-34	42	32	3.93	L	frontal pole
													-40	44	20	4.07	L	frontal pole
							-56	12	2	7.54	L	inferior frontal g., opercular part	-50	6	4	10.22	L	inferior frontal g., opercular part
	-40	-14	46	4.69	L	precentral g. (FEF)	-52	-2	52	5.58	L	precentral g.(FEF)	-36	-4	48	5.81	L	precentral g. (FEF)
	-58	4	26	3.53	L	precentral g.	-58	4	26	3.91	L	precentral g.*	-58	4	26	3.38	L	precentral g.*
**Parietal lobes**	24	-36	76	5.80	R	postcentral g.	22	-32	64	3.47	R	postcentral g.	28	-36	68	4.81	R	postcentral g.
													34	-44	46	5.39	R	sup. parietal lobule, intraparietal sulcus
	52	-56	56	5.49	R	inferior parietal lobule	54	-38	58	3.11	R	inferior parietal lobule	56	-28	44	8.43	R	inferior parietal lobule
	56	-38	38	4.17	R	inferior parietal lobule, supramarginal gyrus	56	-38	38	2.76	R	inferior parietal lobule, supramarginal gyrus*	56	-38	38	4.02	R	inferior parietal lobule, supramarginal gyrus*
	42	-32	28	5.10	R	inferior parietal lobule	46	-34	24	10.6	R	Inferior parietal lobule	48	-32	28	9.58	R	Inferior parietal lobule
	-24	-36	74	6.52	L	postcentral gyrus	-24	-36	70	9.33	L	postcentral gyrus	-26	-38	76	3.74	L	postcentral gyrus
													-32	-50	42	3.83	L	sup.parietal lobule, intraparietal sulcus
	-60	-44	38	3.09	L	inferior parietal lobule, supramarginal gyrus	-62	-36	46	4.76	L	inferior parietal lobule, supramarginal gyrus	-60	-44	38	3.65	L	inferior parietal lobule, supramarginal gyrus*
	-50	-40	24	4.06	L	inferior parietal lobule	-46	-32	22	8.77	L	Inferior parietal lobule	-46	-36	26	8.42	L	Inferior parietal lobule
													16	-68	36	5.75	R	precuneus
													-6	-76	38	3.40	L	precuneus
**Temporal lobes**							52	-52	2	4.68	R	middle temporal g., temporoocipital	60	-58	10	6.13	R	middle temporal g., temporoocipital
							-58	-64	6	5.54	L	middle temporal g., temporoocipital	-56	-68	2	3.61	L	middle temporal g., temporoocipital
							-38	-60	6	8.06	L	temporooccipital, optic radiation	-42	-50	6	4.87	L	temporooccipital, optic radiation
**Occipital lobes**													16	-68	10	3.54	R	calcarine cortex (V1)
													-10	-72	10	3.33	L	calcarine cortex (V1)
**Thalamus**	6	-28	2	5.56	R	thalamus, paramedian	4	-20	10	3.29	R	thalamus, paramedian	8	-16	6	3.59	R	thalamus
							6	-8	14	3.65	R	thalamus, anterior	6	-8	14	3.49	R	thalamus, anterior*
							-2	-16	10	3.24	L	thalamus,paramedian*	-2	-16	10	3.13	L	thalamus, paramedian
	-16	-4	2	4.72	L	thalamus, anterior	-16	-4	2	3.29	L	thalamus, anterior*	-16	-4	2	4.52	L	thalamus, anterior*
**Brain stem**	8	-26	-10	4.66	R	midbrain	8	-24	-14	3.87	R	midbrain	2	-32	-16	6.30	R	midbrain
							-10	-20	-8	3.89	L	midbrain	-12	-18	-8	5.24	L	midbrain
**Cerebellum**	4	-66	-24	4.68	R	midline, vermis VI	-2	-70	-28	7.07	L	midline, vermis VI	2	-66	-30	4.37	R	midline, vermis VI / VII
	-10	-60	-30	7.25	L	dentatus	26	-62	-28	5.63	R	hemisphere V/VI	20	-62	-22	3.80	R	hemisphere V/VI
							-24	-64	-24	5.61	L	hemisphere V/VI	-16	-66	-22	4.17	L	hemisphere V/VI
	-18	-66	-52	6.63	L	hemisphere VIIIa												
	-8	-74	-36	5.94	L	crus II												
**Deactivations**	**PET**						**MRI-block**						**MRI-onset**					
	**x**	**y**	**z**	**T-value**	**Atlas**		**x**	**y**	**z**	**T-value**	**Atlas**		**x**	**y**	**z**	**T-value**	**Atlas**	
**Insula / frontal lobes**	22	22	52	4.52	R	superior frontal g.	24	30	48	6.17	R	sup. frontal gyrus						
	-16	8	52	5.67	L	superior frontal g.												
	2	66	10	5.84	R	frontal pole	14	68	12	4.72	R	frontal pole						
	-16	60	2	7.17	L	frontal pole												
	-28	22	46	6.22	L	middle frontal g.												
							12	36	-8	4.29	R	medial orbitofrontal c.						
**Parietal lobes**	16	-62	58	4.05	R	sup. parietal lobule	12	-54	68	4.65	R	sup. parietal lobule						
	-14	-68	54	4.47	L	sup. parietal lobule	-14	-50	66	4.07	L	sup. parietal lobule						
	42	-56	26	3.71	R	inf. parietal lobule	60	-54	24	5.02	R	inf. parietal lobule						
							42	-72	44	5,53	R	inf.parietal lobule						
	-38	-62	28	3.90	L	inf. parietal lobule	-38	-74	40	3.61	L	inf. parietal lobule						
	46	-22	56	3.94	R	postcentral gyrus	44	-24	68	3.90	R	postcentral gyrus						
	-42	-32	60	8.06	L	postcentral gyrus	-44	-30	58	5.09	L	postcentral gyrus						
**Temporal lobes**	58	-6	-6	4.73	R	superior temporal g.												
	-60	-20	0	5.90	L	superior temporal g.												
	56	-4	-22	4.92	R	middle temporal g.	60	-4	-22	7.27	R	middle temporal g.						
							-50	-8	-20	5.00	L	middle temporal g.						
	26	-18	-22	6.01	R	hippocampus	28	-10	-20	5.03	R	hippocampus						
	28	-32	-10	4.54	R	hippocampus	32	-26	-14	5.08	R	hippocampus						
	-20	-10	-22	5.84	L	hippocampus	-28	-20	-22	3.94	L	hippocampus						
	-26	-32	-2	4.86	L	hippocampus												
**Occipital lobes**	26	-56	-4	4.33	R	lingual gyrus												
	28	-92	6	5.84	R	occipital pole												
	-32	-92	10	6.09	L	occipital pole												
	6	-78	22	6.61	R	cuneus												
	-8	-82	18	4.30	L	cuneus												
	34	-80	16	3.37	R	lateral occipital c.*												

*collected from closest maximum according to other map in same brain area

Results of the statistical group analysis for the contrasts PET vs. rest, BOLD-block vs. rest, BOLD-onset vs. rest, and vice versa (one sample t-test) thresholded at p<0.05 corrected for multiple comparisons (FDR). FEF: Frontal eye field; SMA: supplementary motor area; CSv: cingulate sulcus visual area

#### PET activations

The maximum activation peak was localized in the right anterior insula (9534 voxels), with a corresponding peak in the left anterior insula (2207 voxels). These clusters in both hemispheres extended into the middle insula, and in the right hemisphere further into the inferior frontal gyrus and its opercular part. Further bilateral activations were detected in the inferior parietal lobule in the temporo-parietal junction. A cluster in the midline cingulate/ medial part of the precentral gyrus that is compatible with the location of CSv [67,68] was detected predominantly on the right side, extending across the midline. Thalamic activation maxima were located in the left anterior part, and in the right posterior paramedian part, the latter extending into the midbrain. Activation in the superior frontal gyri (SMA), precentral gyri (FEF), and postcentral gyri (somatosensory) were found bilaterally. The activation with the second highest T-value and cluster size (6471 voxels) appeared in the cerebellum, with its peak in the left dentatus, covering larger parts of the left compared to the right cerebellar hemisphere. Even with sub-threshold (p<0.001 uncorrected) inspection, no activation was found in the posterior insular gyri in PET.

#### BOLD-block activations

Activations in this map were gathered mainly in one single large cluster (49764 voxels). All areas found showed corresponding peak activations bilaterally in both hemispheres. Peak activations were localized bilaterally in the temporo-parietal junction, covering the inferior parietal lobules, retroinsular regions, all insular gyri, the opercula, and extending into inferior frontal gyri. Further activation peaks were found in the middle temporal gyri in the temporo-occipital cortex. Medial activations showed peaks in the precentral gyrus/cingulate sulcus region (CSv). Both thalami and the midbrain were completely covered. Activation in the superior frontal (SMA), precentral (FEF), and postcentral gyri (somatosensory) was also found bilaterally. Midline vermal and bilateral cerebellar hemisphere activations were found in superior parts of the cerebellum. However, we cannot report results below z = -30 mm due to the restricted field of view of the MRI data.

#### BOLD-onset activations

This contrast revealed only one single confluent activation cluster (97553 voxels) in both hemispheres. Locations of peak activations corresponded to the results in the BOLD-block for all areas mentioned above. Additional activation peaks were found in the precuneus and calcarine cortex bilaterally (for details please see Fig 2 and Table 1).

#### PET deactivations

The largest cluster was located in the occipital lobes bilaterally, with its peak deactivation in the precuneus and cuneus, extending to the lateral occipital cortex and the occipital pole. Bilateral postcentral gyri deactivation was found predominantly in the left hemisphere (left: T = 8.06, 827 voxels; right: T = 3.94, 204 voxels). Bilateral hippocampal deactivation was slightly more extended on the right. Further bilateral deactivations were located in the superior temporal gyrus (on the right extending into middle temporal gyrus), in the inferior and superior parietal lobules, the superior frontal gyri, and the frontal poles.

#### BOLD-block negative signals

The largest cluster (3044 voxels) was localized in the precuneus bilaterally with the highest T-value (9.79) on the right, on both sides extending upwards into the superior parietal lobules. Further distinct clusters were found bilaterally in the postcentral gyri (left: 451 voxels, T = 5.09; right: 110 voxel, T = 3.9), the hippocampi (right: 298 voxel, T = 5.08; left: 16 voxel, T = 3.94), the middle temporal gyri, and the inferior parietal lobules (right: 1294 voxel, T = 5.53; left: 87 voxel, T = 3.61). In the right frontal lobe, additional clusters were located in the superior frontal gyrus, frontal pole, and medial orbito-frontal cortex.

#### BOLD-onset negative signals

No clusters were found at the selected threshold, even when the threshold was lowered to p<0.001 uncorrected.

No area was found that was activated in one, and simultaneously deactivated in the other modality or vice versa. The contrasts ‘BOLD-onset x time’ and ‘BOLD-block x time’ gave no significant results.

### Assessment of pairwise differences between PET and MRI

The direct statistical comparison of PET versus BOLD-block (paired t-test) revealed results due to two different situations: higher signal in PET as compared to BOLD-block (right anterior insula, right precentral gyrus, left postcentral gyrus, paramedian thalamus), or activation in PET and no activation in BOLD-block (anterior insula bilaterally, cerebellar dentatus, right caudatus, and right superior frontal gyrus)(S1 Fig and S1 Table).

The direct statistical comparison of BOLD-block versus PET revealed signal differences due to a negative PET signal and close to zero signal in BOLD-block bilaterally in the superior temporal gyri, the hippocampi, and the occipital poles, and unilaterally in left precuneus, left frontal pole, left superior and middle frontal gyrus, left superior parietal lobule, and the right fusiform gyrus. Only in the left postcentral gyrus were signals more negative in PET compared to BOLD-block. A single cluster in the left frontal pole exhibited a positive signal in BOLD-block and close to zero signal in PET.

The direct statistical comparison of PET versus BOLD-onset revealed no significant results. The comparison BOLD-onset versus PET revealed all activations that were found for BOLD-onset itself, and some additional clusters at the locations of deactivations in PET, e.g. in the left occipital pole. Since these clusters are already depicted in Table 1, we will not list the names of the structures again here.

### Assessment of peak locations

**Fig 3:**
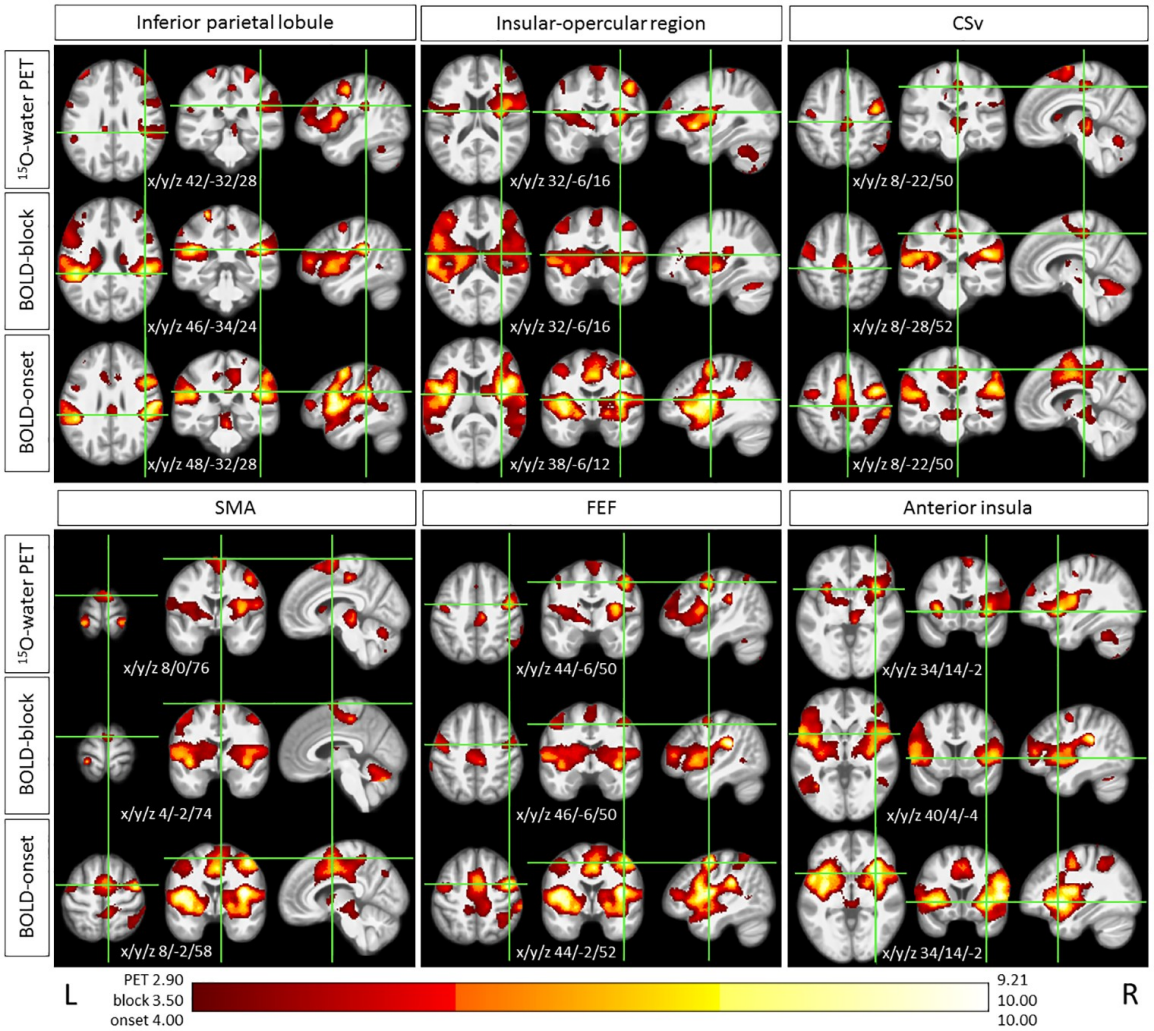
Peak activations during GVS. Example of concordant co-locations of peak activations during GVS in H_2_^15^O-PET, BOLD-block, and BOLD-onset in multisensory vestibular and associated motor areas. The intersections of the green lines on the axial, coronal, and sagittal images equate to the coordinates given in Table 1. For illustrative purposes, the thresholds are adjusted across the modalities. CSv: cingulate sulcus visual area; SMA: supplementary motor areas; FEF: frontal eye-field L: left; R: right.

Comparison of peak activation revealed a proximity in the 3 datasets within known core structures of the vestibular and ocular motor processing network (Table 1, Fig 3). In a few cases of peaks that were not co-located, there were clearly suprathreshold values on the corresponding t-maps of the other modalities. The H_2_^15^O-PET and both BOLD datasets demonstrated in both hemispheres peak activations in the inferior parietal lobules and the anterior insula / insular-opercular region. In the depth of the cingulate sulcus adjacent to the cingulate cortex and the medial part of the precentral gyrus all 3 data sets showed local peak activations on the right, most probably corresponding to the multisensory ego-motion sensitive cingulate sulcus visual area (CSv) [65]. Precentral gyrus activation peaks in the frontal eye field (FEF) [69,70] are also consistently exhibited in all 3 data sets bilaterally. The peaks of activation in the right superior frontal gyrus for all modalities lie within the supplementary motor area (SMA), although the BOLD-onset has a peak 16–18 mm apart from the H_2_^15^O-PET and BOLD-block (Fig 3). PET did not reveal any activation in the temporal lobe, whereas BOLD-onset and BOLD-block did, here mainly in the middle temporal gyrus / temporo-occipital cortex bilaterally. Thalamic activation in PET was restricted to the left anterior part and the right paramedian part, on this side reaching downward to the upper midbrain. In contrast, both MRI activation maps covered the whole thalamus and the complete midbrain bilaterally. Bilateral activation manifested in the superior part of the postcentral gyri in all three modalities. However, in PET and BOLD-block, deactivations in more lateral and inferior parts of the postcentral gyri were found, that were spared in BOLD-onset.

**Fig 4:**
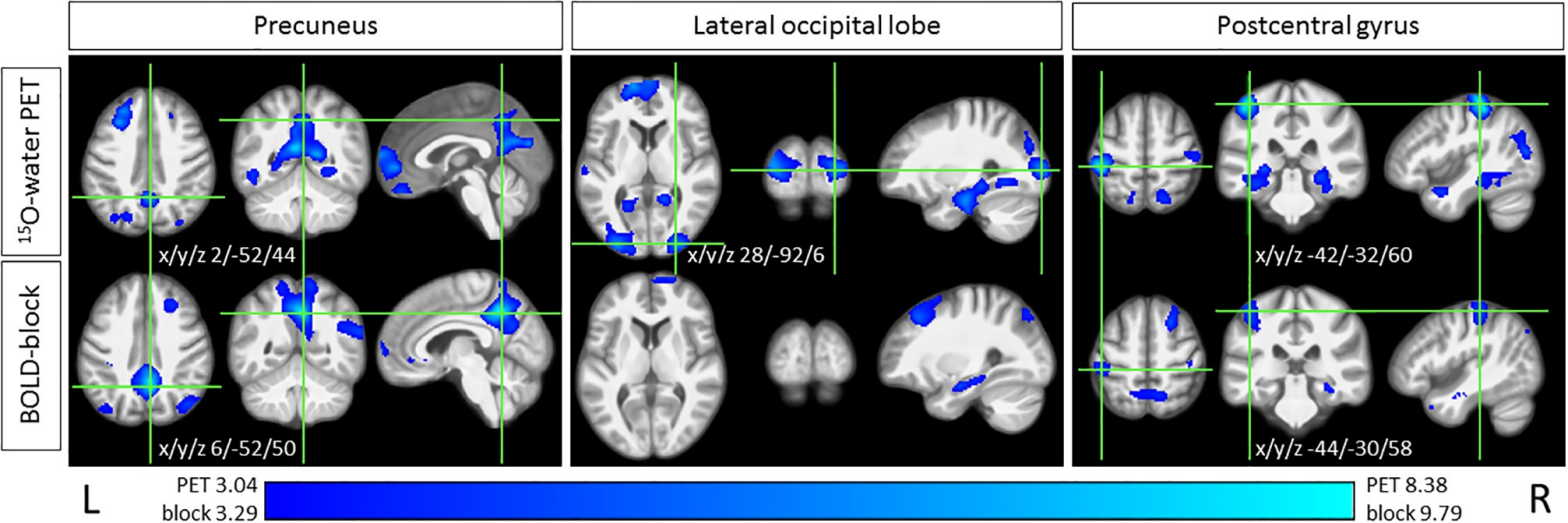
Peak deactivations during GVS. Example of concordant co-locations of peak deactivations during GVS H_2_^15^O-PET and BOLD-block in visual (precuneus) and somatosensory areas (postcentral gyrus). BOLD-onset showed no significant deactivations at all. The intersections of the green lines on the axial, coronal, and sagittal images equate to the coordinates given in Table 1. For illustrative purposes, the thresholds are adjusted across the modalities. L: left; R: right.

Deactivations were seen in PET and BOLD-block, but not at all in BOLD-onset. Concurrent peak deactivations in PET and BOLD-block were located in the precuneus, parts of the inferior and superior parietal lobules, the postcentral gyrus (somatosensory), and the hippocampal formation bilaterally. Deactivation of the superior frontal gyrus /frontal pole manifested bilaterally in PET, but only on the right side in BOLD-block (Table 1, Fig 4). In addition, PET deactivations that were not detected by the other modalities were found in the superior temporal gyri bilaterally, and the visual cortex in the cuneus, occipital pole, and lateral occipital cortex bilaterally.

The contrast estimates as a correlate of the effect strength at certain cluster locations showed no area with a simultaneous significant activation in one contrast and deactivation in the other.

**Fig 5:**
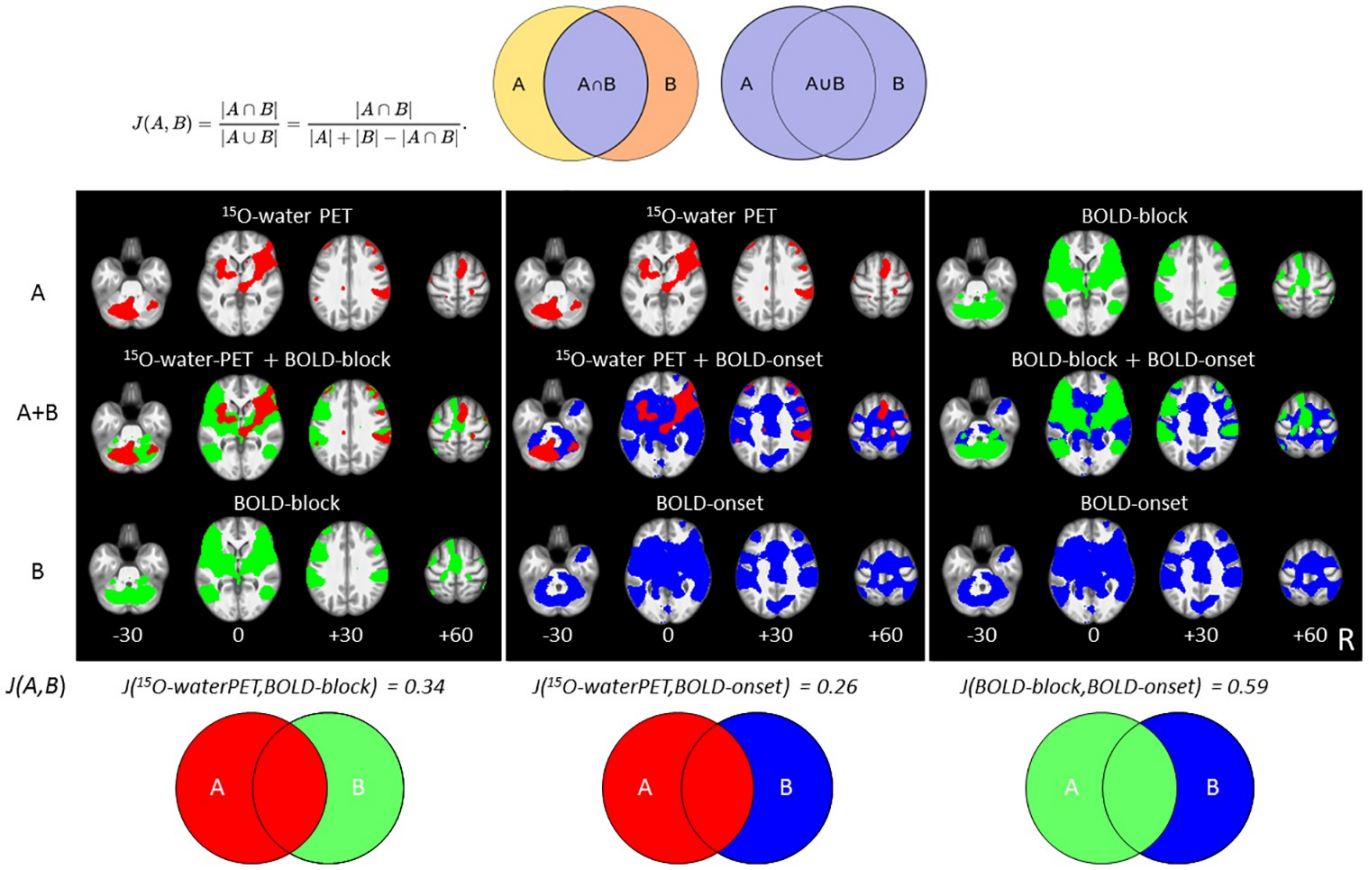
Jaccard similarity coefficient of the different activation data sets. The Jaccard similarity coefficient was applied to compare the extent and overlaps between the 3 activation data sets, H_2_^15^O-PET (red), BOLD-block (green), and BOLD-onset (blue). It is defined as the size of the intersection divided by the size of the union of the sample set (please see formula and illustrative circles) and ranges from zero to one (excellent similarity). In each middle row (A+B), the binary maps of the two modalities are depicted above (A) and below (B). Both BOLD maps show a high similarity (right). Each of the BOLD maps covers the majority of the H_2_^15^O-PET map, but due to different cluster sizes the calculated similarity coefficient is weaker (0.34 and 0.26).

*The Jaccard coefficients*, as a measure for the overlaps between two activation maps, point towards a high similarity between the two BOLD data sets (J = 0.59), a weaker similarity between PET and BOLD-block (J = 0.34) and an even weaker similarity between PET and BOLD-onset (J = 0.26) (Fig 5).

### Lateralization

The laterality index (LI) displays a clear global asymmetry in the PET data set with a right-sided predominance of the activations (LI = -0.53) and weaker left-sided global dominance of the deactivations (LI = 0.25). Unexpectedly, BOLD-block activations were asymmetric towards the left (LI = 0.20), BOLD-block deactivations towards the right hemisphere (LI = -0.58). BOLD-onset shows the least asymmetry of all activation maps, with a slight trend of the activations towards the right hemisphere (LI = -0.12), and has no significant deactivations. The LI in selected brain regions showed the following: for the insular-opercular ROI LI = -0.70/0.10/0.01 for PET/BOLD-block/BOLD-onset respectively, in CSv LI = -0.62/0.35/-0.04, in the superior frontal gyrus (SMA) LI = -0.87/-0.09/-0.11, in FEF LI = -0.53/0.17/-0.19, in the thalamus LI = -0.80/0.21/0.05; for the deactivations LI = -0.05/-0.42 in the precuneus for PET and BOLD-block, respectively. (For visual illustration of the asymmetries see Figs 3 and 4).

## Discussion

This is the first hybrid PET-MR study directly comparing the results of BOLD MRI, which is currently the method most often used for brain activation studies, with those of the gold standard blood flow measurement by H_2_^15^O-PET in an activation paradigm. Binaural galvanic vestibular stimulation was used because it gave sustainable activation patterns across several earlier imaging studies [18,20,50–52,54,68,71], and allows a clear onset of the stimulation to be defined.

**Fig 6:**
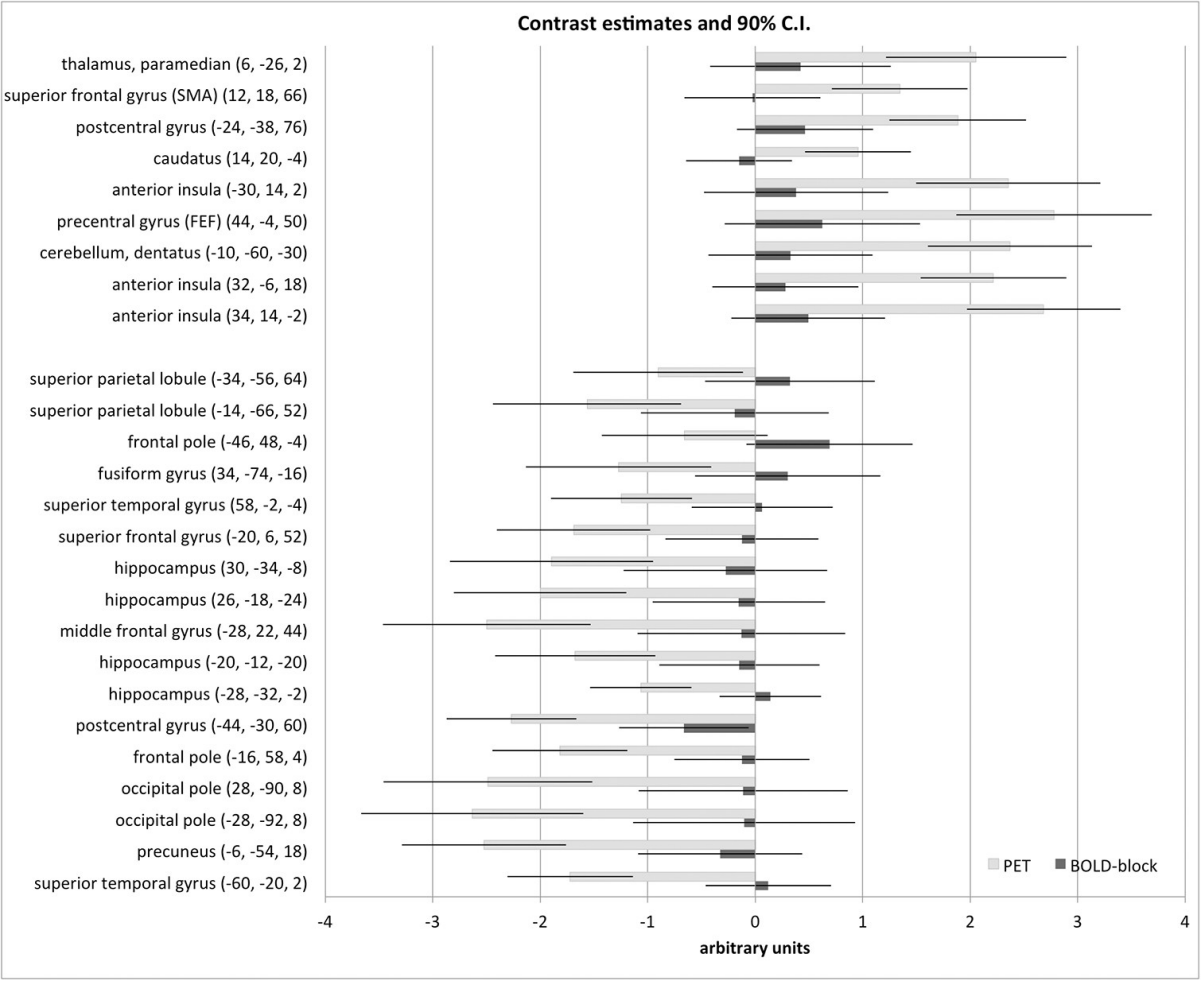
Contrast estimates of peak activations and deactivations. Illustration of the contrast estimates for PET (light gray) and BOLD-block (dark gray) activations (positive values) and deactivations (negative values) for selected peak locations from the paired t-test (S1 Table). The black lines indicate the 90% confidence interval (C.I.). There is no area with a simultaneous significant activation in one contrast and deactivation in the other.

The main results of the present study in healthy volunteers were as follows:

1. For the first time the feasibility of hybrid H_2_^15^O-PET and BOLD MRI scanning in a vestibular stimulation paradigm, e.g. using GVS, was demonstrated.
2. All contrasts used, H_2_^15^O-PET, BOLD-block, and BOLD-onset (event-related) MRI analyses, showed a similar bilateral activation pattern of known cortical vestibular network areas (e.g., the insular-opercular region, inferior parietal lobule, anterior insula), as well as areas linked with specific components of the motor system (e.g., CSv, SMA, FEF) with remarkable proximity of their activation foci (Figs 2–6).
3. BOLD activations (block as well as onset) showed markedly more widespread and bilateral activation than H_2_^15^O-PET at the similar threshold. There were no contradictory signal responses, i.e. no area was found to be activated in one and deactivated in the other contrast. Thus, the MRI activation maps may reflect substantial effects not confirmed by the H_2_^15^O-PET results, which is regarded as gold standard for rCBF measurements (Figs 2 and 6).
4. Whereas PET again confirmed the asymmetric hemispheric activation with clear lateralization to the non-dominant right hemisphere in right-handers, both MRI BOLD contrasts revealed no consistent lateralized pattern: BOLD-onset activation a mild tendency to the right, BOLD-block activation a tendency towards the left hemisphere.
5. PET confirmed concurrent signal decreases (“deactivations”) in the primary and secondary visual cortex areas of the occipital lobe (cuneus, lingual gyrus occipital pole, precuneus), in line with the concept of a reciprocal inhibitory interaction between the visual and vestibular systems. BOLD-onset showed no visual deactivations at all, BOLD-block only decreases in upper secondary visual areas, e.g. in the precuneus, with corresponding peaks compared to H_2_^15^O-PET (Fig 4).
6. Overall, the activation-deactivation pattern in BOLD-block appears to be more similar to the H_2_^15^O-PET than the BOLD-onset.

### Methodological considerations for the comparability of PET and MRI data

Looking at the nature of the signal, PET using H_2_^15^O-PET is a relatively straightforward method measuring the capillary rCBF as a surrogate marker of brain activity [1,3]. In comparison, the BOLD signal is an indirect marker of neuronal activity. It is inherently variable, since BOLD contrast in stimulus-evoked experiments arises owing to a greater increase in cerebral blood flow relative to the cerebral metabolic rate of oxygen, leading to an increase in diamagnetic oxyhemoglobin relative to paramagnetic deoxyhemoglobin in capillaries and veins. A major component of variance arises from complex neurochemical, metabolic, and hemodynamic modulations of cerebral blood flow, blood volume, and blood oxygenation [for review 7]. Depending on the acquisition parameter and experimental design, physiological noise can account for 20–70% of the variance observed in fMRI data collected at 3T [72,73]. Two major sources of physiological noise in the BOLD signal are artifacts induced by the respiration cycle and contractions of the heart [74].

We performed simultaneous PET-MRI scanning. The nature of the two methods leads to some adaptations in the experimental protocol due to differences in the acquisition and statistical analyses (Fig 1). For PET, the rest and stimulation runs are acquired separately, resulting in 3–4 stimulation or rest runs, each including only the signal information from the first 30 seconds at the beginning of each run. Thus, PET does not catch the information from the rest of the stimulation cycle. In contrast, MRI registers the hemodynamic response function of all 4 stimulation and 4 rest blocks within the stimulation runs (each block of 30 seconds duration, in total 260 scans per run). On the other hand, for MRI evaluation we did not take into account the information of the PET rest runs. This difference could hypothetically cause a systematic bias, either by increasing the degrees of freedom for MRI or by increasing the sensitivity for PET relying on separate rest scans. The BOLD signal with alternating stimulation-rest cycles may be influenced by potential arterial volume undershoot during rest immediately after a stimulation [75] or slow venous blood volume recovery [76]. However, each could either increase or decrease the existing signal, but it is unlikely to influence the regional distribution.

A combined PET-MRI study in small laboratory animals found a mismatch in spatial locations [10]. This was explained by a weighting of the BOLD signal contribution towards the venules and venous space, whereas H_2_^15^O-PET reflects changes in CBF, which mainly occur in the arterial space, the capillary bed, and the brain parenchyma [10,77]. Considering these results, it is remarkable how well the activation peaks in the current study co-locate.

Concerning the processing of the data, normalization of global mean values has to be applied to PET data [3] that should not be used for fMRI data. On the other hand, for BOLD data a correction for baseline signal drift of non-experimental factors is performed by removing low frequency components from the data [78]. The PET approach assumes that the global cerebral perfusion remains constant across the stimulation and rest scans. The PET signal compares the activation and the baseline from separate scans, whereas the MRI uses all the on-off data of a run but only from the activation runs (Fig 1). Thus, a potential baseline shift between the runs would only influence the PET data as a systematic bias. On the other side, the MRI baseline in the on-off design may not be a true baseline throughout each run. The differences induced by the non-directly comparable baselines may influence the degree of relative activation and deactivation, but are not likely to influence the regional distribution.

Strong static magnetic fields, such as MRI scanners, themselves induce dizziness and vertigo in human subjects [79], which are caused by inner ear vestibular stimulation mainly of the lateral and anterior semicircular canals by magnetohydrodynamic forces. An accompanying persistent nystagmus diminishes but does not completely disappear over time [80,81]. These artificially induced vestibular imbalance effects must be considered when interpreting MRI activations as well as deactivations to avoid biased results [82]. This is especially true, when comparing separately acquired PET and MRI results. However, in our setup using a hybrid PET-MRI-scanner this effect cannot account for differences between the results of the two contrasts, since the subjects are identically exposed to the magnetic field for both scanning methods.

Thus, these methodological considerations lead us to expect differences between PET and MRI concerning the amount/intensity of activations and deactivations rather than the localization of signal peaks, which is in line with our observed results.

### Activations of the vestibular system

The vestibular system is based on the principle of fusion of bilateral sensors, the input of which is distributed in a bilaterally organized neuronal network [review 16]. The major functions of this system include perceptual, ocular motor, postural, and vegetative functions as well as higher vestibular functions, e.g., navigation and spatial memory. Due to its multisensory nature, e.g., processing not only vestibular, but also visual and somatosensory inputs, the engagement of multiple cortical and subcortical structures appears to be a logical consequence. In fact, functional imaging studies applying variable designs and vestibular stimuli, e.g., caloric, galvanic, or sound-evoked vestibular stimulation, consistently identified a bihemispherical network, in separate and distinct mainly temporo-parietal areas [14]. Areas in the right posterior insular-opercular region are generally supposed to represent the human homologue of the multisensory vestibular cortex (PIVC) in monkeys [23–25,83].

In our study, H_2_^15^O-PET and both BOLD MRI contrasts showed corresponding bilateral peak activations in this core region, but also close proximity of maxima in other cortical vestibular network areas, e.g. in the inferior parietal lobule, inferior frontal gyrus, and the anterior insula, as well as in areas linked with specific components of the motor system, in particular the cingulate sulcus visual area (CSv), the supplementary motor area (SMA), and the frontal eye fields (FEF) (Fig 3, Table 1). The area CSv is thought to be closely involved in encoding egomotion, which is also introduced by GVS in terms of a sensation of being tilted or nudged. It responds not only selectively to visual coherent optic flow, but also receives strong vestibular afferents [67,68], and provides the sensory information further to the motor system for use in guiding locomotion, e.g. by strong connectivity with the supplementary motor area (SMA) [65]. The latter contributes to the control of movement and thereby postural stabilization of the body. The FEF is known to be involved in a variety of ocular motor tasks including the execution of vestibular evoked eye movements, also mildly induced by GVS [44,69,84].

Potentially relevant discrepancies within the vestibular network were found for the thalamus-midbrain level: at the given threshold H_2_^15^O-PET shows circumscribed activations only of the right posterior and paramedian part of the thalamus (the latter merging down to the midbrain), whereas BOLD activation covers more or less the complete thalami and midbrain area on both sides, but with low z-scores and with peak activations only in the anteromedial part (Fig 2). This discrepancy might be caused by intravascular overestimation near the major arteries at brainstem level in MRI, e.g. the basilary artery and the Circulus arteriosus Willisi and its outflows [10,13].

In summary, the reliable bilateral cortical activation pattern with correlating maxima in H_2_^15^O-PET, BOLD-block and BOLD-onset provided a good basis for further comparative analyses regarding lateralization effects.

#### Signal decreases during vestibular stimulation

Accompanying relative signal decreases (“deactivations”) within the visual and somatosensory cortices have been repetitively and consistently reported in PET as well as in earlier MRI studies on the vestibular system at 1.5T [14,28,39,40,51], even though the origin of negative responses and their relationship to neuronal activity is not fully understood, especially for negative BOLD MRI signals. However, a study combining functional MRI and electrophysiological recording demonstrated a close correlation between negative BOLD responses beyond stimulated regions of the visual cortex and local neuronal activity decreases [85]. The functional interaction between the vestibular and visual systems was further underlined by a psychophysical study that showed impaired visual mental rotation during caloric vestibular stimulation [86]. Due to their systematic appearance at comparable sites in the visual and somatosensory cortex with different imaging modalities, e.g., BOLD MRI, FDG-PET, H_2_^15^O-PET, in healthy subjects and vestibular patients, a functional relevance appeared very likely, and led to the concept of a reciprocal inhibitory cortical interaction between sensory systems, e.g. the vestibular and visual systems [40,41]. This interaction provides a powerful means for shifting the dominant sensorial weight from one modality to the other for resolving conflicts between incongruent sensory inputs [19].

In the current study, only PET revealed bilateral deactivations in the *primary* visual occipital cortex (lateral occipital cortex, occipital pole, cuneus, lingual gyrus) during GVS. In line with the reciprocal inhibitory interaction concept, PET and BOLD-block both found signal decreases in upper *secondary* visual areas in the precuneus (Fig 4). However, the complete lack of visual cortex deactivation in BOLD-onset is an interesting finding and might argue for a time-based staggering of the inhibitory interaction pattern. In fact, in the PET-literature, visual deactivations have been mainly described in studies either applying longer vestibular stimulation, e.g., caloric irrigation in healthy subjects [H_2_^15^O-PET: 30,31,87], or investigated patients with ongoing vertigo and vestibular tone imbalance, e.g. due to an acute unilateral vestibulopathy [FDG-PET: 35,88,89]. The majority of MRI studies reporting visual cortex deactivation, all applied blockwise analyses at 1.5 T [GVS: 20,51,53; VEMPs: 21,38,90]. To the best of our knowledge there is only one study available that used an event-related MRI approach with GVS before, but did not analyze negative responses [52]. In the few studies available calculating signal decreases during unilateral VEMP stimulation short tone burst induced otolith stimulation was continuously applied with a repetition rate of 2.5–3 Hz during the stimulation blocks, which might lead to an ongoing central vestibular effect that also results in an inhibitory interaction pattern, although the stimulus per se is very short [21,38,90]. A recent 3 T MRI independent component analyses study during VEMP stimulation in fact detected negative correlations or decreased event-related BOLD-responses as part of the reciprocal inhibitory visual-vestibular interaction [91].

Since the skin was locally anesthetized with lidocaine gel and the subjects perceived no pain or somatosensory sensation, it appears unlikely that the deactivation of the postcentral gyrus bilaterally is caused by skin effects, but rather by the reciprocal inhibitory cortical interaction, in the sense of a more general way of the human brain solving intersensory conflicts. Furthermore, skin irritation would lead one to expect activation and not deactivation of the somatosensory cortex.

Further interesting deactivations were found in PET and BOLD-block at comparable sites in the hippocampal formation bilaterally. Self-motion information provided by the vestibular system to hippocampal formation plays an important role in the development of spatial memory and adequate spatial navigation [92]. Many studies over the last two decades have shown that selective activation of the vestibular system, using either natural rotational or translational stimulation, or electrical stimulation of the peripheral vestibular system, can induce and modulate theta activity [93,94]. One can speculate that hippocampi are “deactivated” in the present study due to the artificial nature of the stimulus providing no useful vestibular information for higher cognitive processes. However, the literature on hippocampal/parahippocampal signal changes in vestibular MRI is inconclusive, partly showing no effect, partly activations [caloric: 38; VEMP: 95], but in the majority deactivations [GVS: 20; caloric: 28; VEMPs: 21,87,90].

#### Similarities and discrepancies in the activation pattern

In general, as expected the BOLD MRI data display a much more widespread signal than PET, especially BOLD-onset. Consequently, the Jaccard coefficients, as a measure for the overlaps between two activation maps, pointed towards a high similarity between the two BOLD data sets (J = 0.59), weaker similarity between PET and BOLD-block (J = 0.34), and low similarity between PET and BOLD-onset (J = 0.26) (Fig 5). One explanation for the relatively low similarity index between PET and BOLD could be a smaller number of significant PET voxels and larger number of significant BOLD voxels at the given threshold.

Since differences are most probably signal intrinsic and hampered evaluation of direct statistical comparison of the different contrasts, we compared the cluster maxima of all three contrasts, which showed a remarkable proximity despite the methodological disparities discussed above (Table 1). Furthermore, no area was found to be activated in one contrast and deactivated in the other. In a nutshell, although H_2_^15^O-PET and MRI activation maps (Figs 2 and 6) exhibit remarkably similar activation peaks, there are still differences that cannot be explained by a simple thresholding effect. Furthermore, the activation-deactivation pattern of the BOLD-block is more similar to the rCBF PET than that of BOLD-onset. From a neurological functional point of view, the information of H_2_^15^O-PET and BOLD-block appears comparable, providing equally relevant information concerning activation peaks.

However, the MRI activation maps may reflect additional substantial effects not confirmed in the H_2_^15^O-PET results, which is regarded as the gold standard for rCBF measurements.

### Lateralization aspects

The asymmetry index reveals the most substantial differences, justifying further reflection. The H_2_^15^O-PET displayed a clear dominance of activation in the non-dominant right hemisphere (right-sided LI = -0.53; left-sided LI 0.25) that is in line with earlier structural and functional human imaging studies [14,16,23,27–29]. The physiological relevance of hemispheric dominance has also been proven by non-imaging methods using transcranial direct current stimulation (tDCS) [96]. In contrast, the present BOLD MRI data showed no consistent lateralized activation pattern, especially in the BOLD-block it was more bilateral and symmetric (LI BOLD-block = 0.20; LI BOLD-onset = -0.12). One might argue that the laterality issue might be influenced by the fact that 2 out of our 19 subjects were left-handed; however, a significant effect is not to be expected, especially not between the two imaging methods applied in identical subjects.

Hemispherical dominance was most convincing in activation studies using caloric [25,28,31,37,52,80,96] or VEMP stimulation [21,38,90]. MRI studies using GVS with varying paradigms consistently reported bilateral activations [18,20,50–54,68] except for two studies, both referring to small sample sizes (Fink et al. 2003: n = 6; Smith et al. 2012: n = 9) [68,71]. However, none of the GVS MRI studies cited calculated hemispherical differences statistically. In the present study, GVS is for the first time measured using H_2_^15^O-PET and again revealed a clear right-hemispherical dominance of the vestibular cortical system, which argues against the earlier hypothesis of a purely stimulus-dependent explanation for the bilateral effects of GVS seen in MRI due to sinusoidal and/or bipolar application.

So why is there a consistent discrepancy between rCBF PET and BOLD MRI during GVS? The reason could be either technical, how the data are acquired and processed, or alternatively/additionally biological-physiological, because the nature of the signal measured is different to vestibular caloric or sound-evoked stimulation.

The vestibular system is thought to play a role in postural-related adjustments of blood pressure and vasoconstriction, which require a decrease of parasympathetic activity and an increase of sympathetic activity during orthostatic challenge (for review, see Carter and Ray 2008) [97]. Even GVS induces a robust modulation of the vestibulosympathic reflex via otolith activation and has been shown to be involved in rapid regulation of arterial blood pressure [98,99]. In contrast, activation of the semicircular canals via caloric stimulation [100–102] or sinusoidal yaw head rotation [103,104] appear to not that consistently alter sympathetic outflow measured mainly by muscle sympathetic nerve activity [97]. A modulation of the vestibulosympathic reflex by GVS would induce rapid physiological changes that are no longer limited to a specific brain hemisphere, resulting in a more symmetrical bilateral pattern as seen in the present study interpreted as a BOLD effect, but not in rCBF PET. As the subjective effect is reported to be strongest at onset of GVS and the vestibulosympathic reflex is a rapidly responding system, it is also in agreement with a more prominent induction of physiological noise with the event-related HFR, here the BOLD-onset. High-pass filter and modeling of the HFR will normally filter out random physiological noise, but in our case, the changes are inherently tied to the stimulation and might therefore influence the results. It is reported that this kind of physiological noise occurs predominately along the major blood vessels areas and their perivascular spaces [105] and parts of the BOLD-onset signal in our study are astonishingly similar to the distribution of some of the major cerebral arteries, e.g. in the brainstem-thalamic regions.

### Limitations and perspectives

There is a rising awareness in the MRI-community that physiological noise, especially induced by the respiration cycle and contractions of the heart, may be included in the statistical analysis and falsely interpreted as BOLD. If these changes are task-related, the BOLD signal can be corrupted through B0 field modulations, T1 inflow and pulsatile motion and direct modulation of blood oxygenation itself [74]. Systematic heart rate changes known to occur in many sensory and motor control tasks as well as during feedback processing in cognitive tasks can result in artificial changes of the BOLD signal caused by secondary physiological processes induced by the task. Such secondary changes in physiological parameters could also be induced by GVS. So, one limitation of this study is that no respiratory, cardiac, or pulse oxygenation monitoring took place during scanning to perform corrections. However, these methods are not routinely established, and would further complicate the already complex set-up for scanning. However, it would be an interesting approach in order to validate whether it is possible to come closer to a purer BOLD signal related to rCBF and neuronal activity. Another promising approach could be to use the newly established and non-BOLD dependent method of arterial spin labeling (ASL) [2,106] and/or to calculate quantitative values from PET by using invasive arterial sampling for the input function.

## Conclusions

For first time, the feasibility of hybrid H_2_^15^O-PET and BOLD MRI scanning was demonstrated in a vestibular stimulation paradigm. All contrasts used, H_2_^15^O-PET, BOLD-block and BOLD-onset MRI, demonstrated a remarkable proximity of their activation foci. PET confirmed the hemispheric laterality aspects and visual-vestibular interaction pattern. BOLD-block appears more similar to PET than BOLD-onset. However, both BOLD MRI activations showed markedly more wide-spread and symmetric activation than H_2_^15^O-PET. Thus, the MRI activation maps may reflect substantial effects not confirmed in the H_2_^15^O-PET results, and may not be regarded as directly rCBF-related.

## Supporting information

S1 Fig Direct comparison of PET versus BOLD MRI. Results of the direct statistical comparison for the contrasts BOLD-block vs. PET as well as BOLD-onset vs. PET, and vice versa (paired t-test) thresholded at p<0.05 corrected for multiple comparisons (FDR) superimposed onto mean T1 image of the subject group. Stronger responses in MRI compared to PET are indicated in red, stronger PET responses compared to MRI are indicated in blue.
(TIF)

S1 Table Direct comparison of PET versus BOLD-block. Results of the direct statistical comparison for the contrasts BOLD-block vs. PET, and vice versa (paired t-test) thresholded at p<0.05 corrected for multiple comparisons (FDR).
(DOCX)
