# Supplementary figures and images for: Direct comparison of activation maps during galvanic vestibular stimulation: A hybrid H2[15 O] PET—BOLD MRI activation study
Source: PLoS One. 2020 May 15;15(5):e0233262. doi: 10.1371/journal.pone.0233262 (PMC7228124; doi:10.1371/journal.pone.0233262)

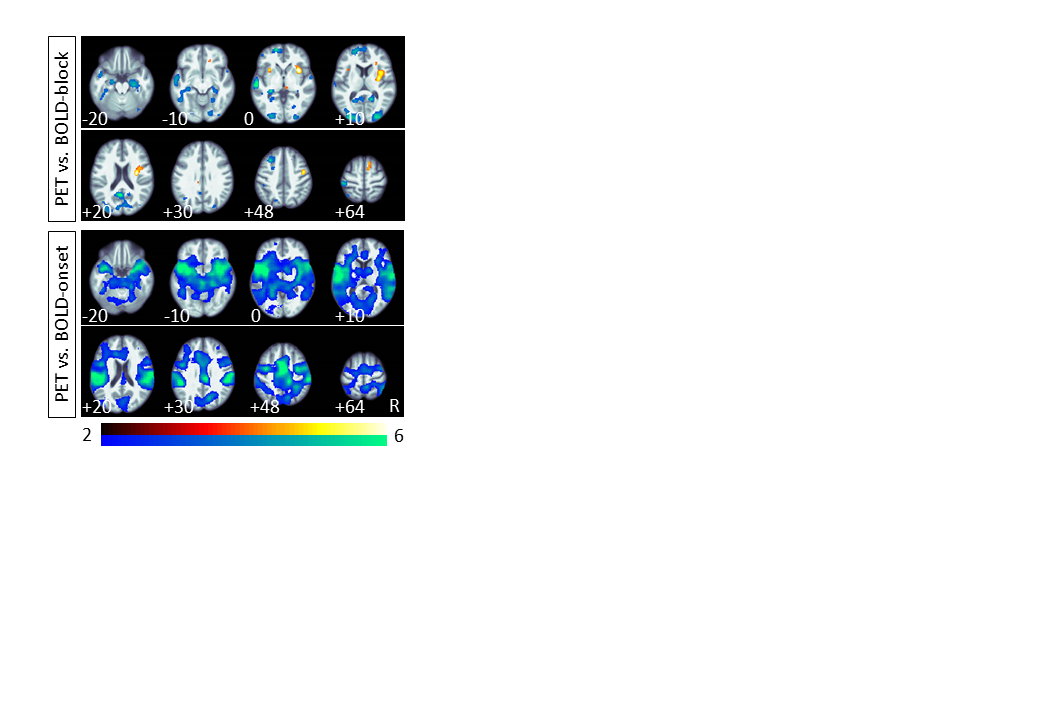

Supplement: S1 Fig — Results of the direct statistical comparison for the contrasts BOLD-block vs. PET as well as BOLD-onset vs. PET, and vice versa (paired t-test) thresholded at p<0.05 corrected for multiple comparisons (FDR) superimposed onto mean T1 image of the subject group. Stronger responses in MRI compared to PET are indicated in red, stronger PET responses compared to MRI are indicated in blue. (TIF) [file pone.0233262.s001.tif]
