# Supplementary material for: Direct comparison of activation maps during galvanic vestibular stimulation: A hybrid H2[15 O] PET—BOLD MRI activation study
Source: PLoS One. 2020 May 15;15(5):e0233262. doi: 10.1371/journal.pone.0233262 (PMC7228124; doi:10.1371/journal.pone.0233262)
Supplement: S1 Table — Results of the direct statistical comparison for the contrasts BOLD-block vs. PET, and vice versa (paired t-test) thresholded at p<0.05 corrected for multiple comparisons (FDR). (DOCX) [file pone.0233262.s002.docx]

| **BOLD-block vs. PET (FDR 0.05)** | | | | | |
| --- | --- | --- | --- | --- | --- |
| **x** | **y** | **z** | **T-value** |  | **Atlas** |
| -60 | -20 | 2 | 7.34 | L | superior temporal gyrus |
| -6 | -54 | 18 | 6.68 | L | precuneus |
| -28 | -92 | 8 | 5.70 | L | occipital pole |
| 28 | -90 | 8 | 5.69 | R | occipital pole |
| -16 | 58 | 4 | 6.28 | L | frontal pole |
| -44 | -30 | 60 | 6.18 | L | postcentral gyrus |
| -28 | -32 | -2 | 5.94 | L | hippocampus |
| -20 | -12 | -20 | 4.76 | L | hippocampus |
| -28 | 22 | 44 | 5.71 | L | middle frontal gyrus |
| 26 | -18 | -24 | 5.35 | R | hippocampus |
| 30 | -34 | -8 | 3.98 | R | hippocampus |
| -20 | 6 | 52 | 5.11 | L | superior frontal gyrus |
| 58 | -2 | -4 | 4.63 | R | superior temporal gyrus |
| 34 | -74 | -16 | 4.25 | R | fusiform gyrus |
| -46 | 48 | -4 | 4.05 | L | frontal pole |
| -14 | -66 | 52 | 3.65 | L | superior parietal lobule |
| -34 | -56 | 64 | 3.62 | L | superior parietal lobule |
| **PET vs. BOLD-block (FDR 0.05)** | | | | | |
| 34 | 14 | -2 | 7.12 | R | anterior insula |
| 32 | -6 | 18 | 6.63 | R | anterior insula |
| -10 | -60 | -30 | 6.22 | L | cerebellum, dentatus |
| 44 | -4 | 50 | 5.51 | R | precentral gyrus (FEF) |
| -30 | 14 | 2 | 5.36 | L | anterior insula |
| 14 | 20 | -4 | 5.22 | R | caudatus |
| -24 | -38 | 76 | 5.20 | L | postcentral gyrus |
| 12 | 18 | 66 | 5.05 | R | superior frontal gyrus (SMA) |
| 6 | -26 | 2 | 4.53 | R | thalamus, paramedian |
